# Phylogenomics Resolves the Phylogeny of Theaceae by Using Low-Copy and Multi-Copy Nuclear Gene Makers and Uncovers a Fast Radiation Event Contributing to Tea Plants Diversity

**DOI:** 10.3390/biology11071007

**Published:** 2022-07-04

**Authors:** Lin Cheng, Mengge Li, Qunwei Han, Zhen Qiao, Yanlin Hao, Tiago Santana Balbuena, Yiyong Zhao

**Affiliations:** 1 Henan International Joint Laboratory of Tea-Oil Tree Biology and High Value Utilization, Xinyang Normal University, Xinyang 464000, China; lzc5569@xynu.edu.cn (L.C.); menggeli1@163.com (M.L.); hanqw1997@163.com (Q.H.); qiaozhen202228@163.com (Z.Q.); haoyl1997@163.com (Y.H.); 2 Department of Agricultural, Livestock and Environmental Biotechnology, Sao Paulo State University, Jaboticabal 14884-900, Brazil; tiago.balbuena@unesp.br; 3 State Key Laboratory of Genetic Engineering, Collaborative Innovation Center of Genetics and Development, School of Life Sciences, Fudan University, Shanghai 200433, China; 4 College of Agriculture, Guizhou University, Guiyang 550025, China

**Keywords:** Theaceae, tea family, *Camellia*, nuclear phylogeny, divergence time estimation, diversification, speciation, fast radiation

## Abstract

**Simple Summary:**

The Theaceae includes more than 300 species of great morphological diversity and has immense economic, cultural, and ornamental values. However, the evolutionary history of this family remains elusive. We integrated 91 genomes and transcriptome datasets of Theaceae and successfully resolved the phylogeny of Theaceae including relatives of cultivated tea plants from both extensive low-copy and multi-copy nuclear gene markers. Bayes-based molecular dating revealed that the ancestor of the tea family originated slightly earlier than the K-Pg boundary (Mass extinction events including the extinction of dinosaurs) with early diversification of three tribes associated with the Early Eocene Climatic Optimum. Further speciation analysis suggested a sole significant diversification shift rate in the common ancestor of *Camellia* associated with the Mid-Miocene Climatic Optimum. Collectively, polyploidy events, and key morphological innovation characters, such as pericarp with seed coat hardening, could possibly contribute to the Theaceae species diversity.

**Abstract:**

Tea is one of the three most popular nonalcoholic beverages globally and has extremely high economic and cultural value. Currently, the classification, taxonomy, and evolutionary history of the tea family are largely elusive, including phylogeny, divergence, speciation, and diversity. For understanding the evolutionary history and dynamics of species diversity in Theaceae, a robust phylogenetic framework based on 1785 low-copy and 79,103 multi-copy nuclear genes from 91 tea plant genomes and transcriptome datasets had been reconstructed. Our results maximumly supported that the tribes Stewartieae and Gordonieae are successive sister groups to the tribe Theeae from both coalescent and super matrix ML tree analyses. Moreover, in the most evolved tribe, Theeae, the monophyletic genera *Pyrenaria*, *Apterosperma,* and *Polyspora* are the successive sister groups of *Camellia*. We also yield a well-resolved relationship of *Camellia*, which contains the vast majority of Theaceae species richness. Molecular dating suggests that Theaceae originated in the late L-Cretaceous, with subsequent early radiation under the Early Eocene Climatic Optimal (EECO) for the three tribes. A diversification rate shift was detected in the common ancestors of *Camellia* with subsequent acceleration in speciation rate under the climate optimum in the early Miocene. These results provide a phylogenetic framework and new insights into factors that likely have contributed to the survival of Theaceae, especially a successful radiation event of genus *Camellia* members to subtropic/tropic regions. These novel findings will facilitate the efficient conservation and utilization of germplasm resources for breeding cultivated tea and oil-tea. Collectively, these results provide a foundation for further morphological and functional evolutionary analyses across Theaceae.

## 1. Introduction 

Theaceae is one of the most well-known and diverse plant families, with nearly 372 accepted species and many important ecological, horticultural, and economic members, including tea plant (*Camellia sinensis* (L.) Kuntze), oil-tea plant (*Camellia oleifera* Abel), and some woody ornamental species such as *Camellia japonica, Camellia sasanqua,* and *Camellia reticulata* [1] (WFO 2021). The tea plant is one of the most significant and traditional economic crops grown in Asia, Africa, and Latin America, whose leaves are used to produce numerous kinds of tea [2,3,4]. *Camellia oleifera* is a woody oil plant, whose seed kernels produce abundant edible oils with high monounsaturated fatty acid content [5,6,7]. The planting area for *C. oleifera* was about 4.39 million hectares in China with a total output value of 116 billion RMB [6]. It has been utilized extensively in France, Japan, and the USA, as a source of additive for cosmetics [8]. Some other ornamental species, such as *Camellia japonica* and *Camellia sasanqua,* are the most well-known *Camellia* plants because of their aesthetic appeal and ideal characteristics as landscape plants. Many cultivated varieties of *C. japonica* and *C. sasanqua* produce colorful flowers with striking aromas [9].

Theaceae, which belongs to Ericales, has recently been delineated into three tribes and nine genera [10]. The classification of the Theaceae is challenging due to its similar morphological characters in some species, prevalence of self-incompatibility, frequent interspecific hybridization, and polyploidization [11]. At the tribe level, the systematics of Theaceae have been analyzed mainly using plastid sequences, including studies with extensive taxon sampling representing most genera, albeit with only two to ten plastid sequences [12,13]. The phylogeny of Theaceae with 30–46 species has also been inferred using a combination of one plastid, one mitochondrial and one nuclear sequence [14], one mitochondrial gene [15], or ten chloroplast sequences plus the nuclear internal transcribed spacer (ITS) [13]. In addition, the plastome phylogenomics method was used to infer relationships among the Theaceae genera [16,17,18]. Recent analyses of Theaceae phylogeny have also been conducted with 610 nuclear genes from 57 species [19]. However, conflicts or poorly resolved relationships still remain among tribes, particularly among the genera and subgenus. Analyses using either plastid genes or DNA Internal transcribed spacer (ITS) indicated that tribes Stewartieae and Gordineae are successive sisters to tribe Theeae [13,14,20]. The same result was also shown in two studies by using plastome data [16,18]. There was weak support for phylogenetic relationships among the three tribes based on the plastid datasets. Organellar genes are generally inherited uniparentally, and the recombination and gene conversion in the plastid genome might also cause biases and errors in reconstructing their phylogenies. 

The phylogenetic resolution among genera is limited by plastid gene makers as well.

*Camellia* is the largest genus in the Theaceae family, and is distributed in China and its adjacent countries. Southern China is a center of diversity of many genera of Theaceae, and also represents an area of endemism and the main massing of *Camellia* in a pan-biogeographic sense [21]. A well-resolved phylogeny is a framework to facilitate the understanding of the origin and morphological evolutionary patterns for these cultivated and economic groups such as the genus of *Camellia*. However, conflicts or poorly resolved relationships still remain among several tribes or genera, particularly among the subgenera within the *Camellia* [22]. The controversy includes two aspects: the contradiction of the relationships based on morphological classification and the evolutionary analysis based on molecular information. 

More recently, nuclear genes have been successfully used to resolve relationships among the deep angiosperm lineages and within orders and families [23,24,25]. Numerous effective nuclear genes could provide alternative data for resolving the reticulated relationship because it is biparentally inherited with more informative sites [25]. With the development of high-throughput sequencing technologies, phylogenomics and phylotranscriptomics have become effective methods for evolutionary analyses in plants. Nuclear gene sequences can be acquired cost-effectively from non-model species, as recently applied in studies of Theaceae [2,3,4,5,6,7,19,25,26,27,28,29,30,31]. The relationship in Theaceae has been analyzed in 610 nuclear genes from 57 species to reconstruct the phylogeny with combined supermatrix and coalescent tree inference methods [19]. These results supported the monophyly of Theaceae; Stewartieae was resolved as sister to the other two tribes. Within Theeae, the *Apterosperma*-*Laplacea* clade grouped with *Pyrenaria*, leaving *Camellia* and *Polyspora* as a sister group. Fifty-seven representative species of Theaceae, as well as additional plastome sequence data, were generated [19,25].

In this study, genomes and transcriptomes of 91 Theaceae species were integrated, covering three tribes and eight genera. In addition, the topology here includes well-supported relationships among eight genera and some important subgenus. We also present well-resolved relationships within *Camellia*, which contains the vast majority of representatives of Theaceae. Molecular dating and speciation rate calculation revealed a fast radiation event in the ancestor of *Camellia* nearly 25 million years ago. Genome polyploidizations, morphological innovation, and suitable geological climates possibly collectively contributed to the diversification of the tea family and helped it survive the mass extinction event. The results provide a strong foundation for further evolutionary studies of Theaceae, contributing to a better understanding of this important group with significant contributions to tropical and subtropical ecosystems. 

## 2. Materials and Methods

### 2.1. Data Source and Transcriptome Assembly

A total of 128 public datasets were collected in our study, including 44 genomic data, 81 transcriptome data, and three genome-skimming data. For detailed source information for these species, please refer to Supplementary Table S1. In our study, 37 species were selected as outgroups, including basal angiosperms, Magnolias, Monocotyledons, early-diverging eudicotyledons, and Ericales. Within Ericales, the public genome representative species from Actinidiaceae, Ericaceae, Primulaceae, Clethraceae, Ebenaceae, and Roridulaceae were curated as closed outgroups of Theaceae. There are 91 public transcriptome and genome-skimming data of Theaceae, including 71 of tribe (Tr.) Theeae, nine of Tr. Gordonieae, and 11 of Tr. Stewartieae, covering eight genera of Theaceae. Genome data were mainly downloaded from the website Phytozome (https://phytozome-next.jgi.doe.gov/ (accessed on 25 August 2021)), while transcriptome datasets were mainly downloaded from NCBI (National Center for Biotechnology Information, https://www.ncbi.nlm.nih.gov/ (accessed on 3 November 2021)).

To obtain high-quality sequencing data, we filtered all transcriptome data using Trimmomatic v.0.39 [32], removing bases with a mass of less than 10 at the head and tail of each read, filtering out bases with an average mass of less than 20 using a sliding window of size four bp, and finally, reads with a length of less than 36 bp were also deleted. The specific parameter command is “LEADING:10 TRAILING:10 SLIDINGWINDOW:4:20 MINLEN:36” for Trimmomatic quality control process. All transcriptomes were de novo assembled into contigs using Trinity v2.11.0 [33]. TransDecoder v5.5.0 (http://transdecoder.sourceforge.net/ (accessed on 8 November 2021)) was used to predict CDS regions. Redundant contigs from each sample were reduced using CD-HIT 4.8.1 [34] with the parameter “-c 0.98” as described in the previous studies [35,36,37,38]. BUSCO v5.2.2 was used to evaluate the quality of the de-redundant CDS sequence, and the comparison database is eudicots_odb10 [39].

### 2.2. Orthologs Identification and Gene Sets Filtrations

Orthologous genes (OGs) were identified with OrthoFinder v2.0.0 (http://www.stevekellylab.com/software/orthofinder (accessed on 8 November 2021))) through 11 species of representing 8 families (*Lactuca sativa* [40], *Chrysanthemum seticuspe* [41], *Ducus carota* [42], *Solanum lycopersicum* [43], *Capsicum annuum* [44], *Camellia sinensis* var. *sinensis* ‘Shuchazao’ [2], *Camellia sinensis* var. *assamica* ‘Yunkang 10′ [26], *Actinidia chinensis* [45], *Primula veris* [46], *Vitis vinifera* [47], and *Aquilegia coerulea* [48]).

**Figure 1:**
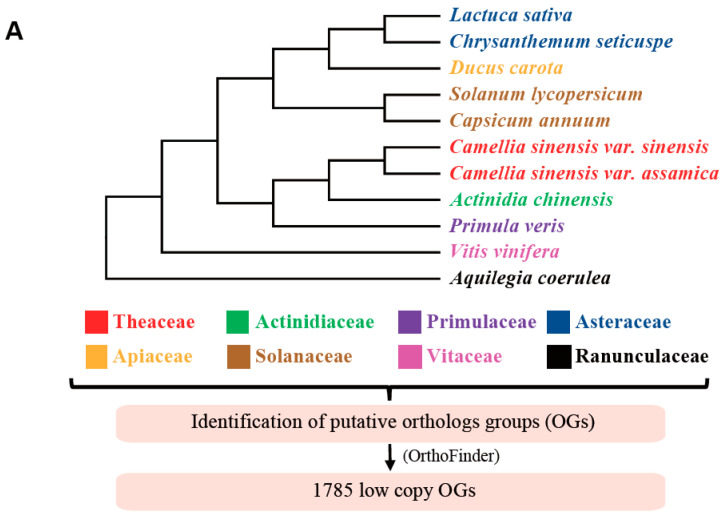

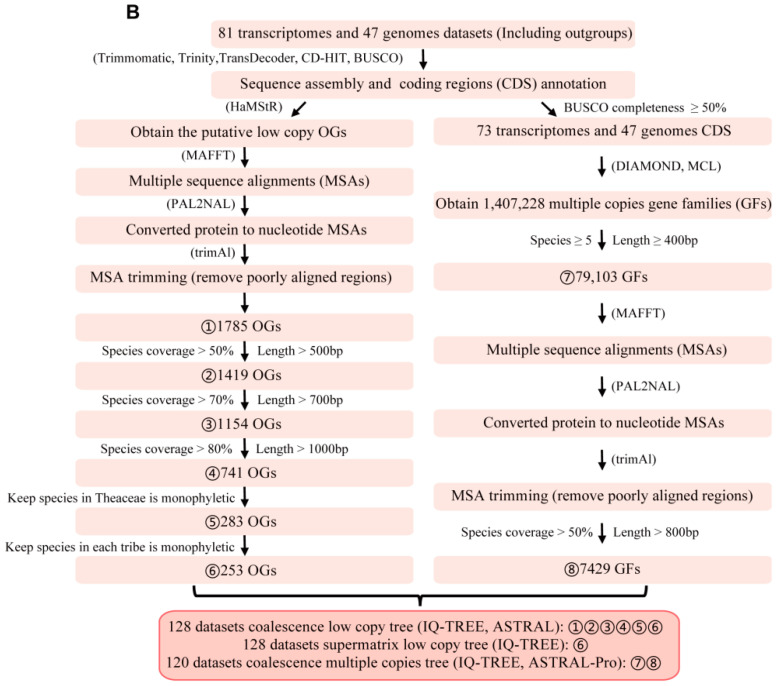
The flowchart of identification and filtration of low-copy nuclear gene sets: (**A**) The flowchart for the identification of low-copy orthologous groups. (**B**) The technical flowchart of phylogenetic tree reconstruction and nuclear genes filtration in Theaceae.

The resulting 1785 OGs were used as seed genes to obtain the corresponding putative orthologs (E-value < 1 × 10^−20^) from 128 samples in HaMStR v13.2.6 [49]. Subsequently, 1785 OGs (set 1) were selected (Figure 1), aligned using MAFFT v7.487 [50] with default settings, and trimmed using trimAl v1.2 [51] with default settings. Next, additional filtering based on taxon coverage, alignment length, and other parameters yielded five smaller sets (sets 2 to 6) of 1419 to 253 OGs (Figure 1). Then, the sequences with relatively low taxon coverage and short alignment regions were removed to obtain successively smaller gene sets, which effectively reduce noise and errors, and facilitate the reconstruction of a robust phylogeny from coalescent analyses.

### 2.3. Gene Ontology Analyses

**Figure 2:**
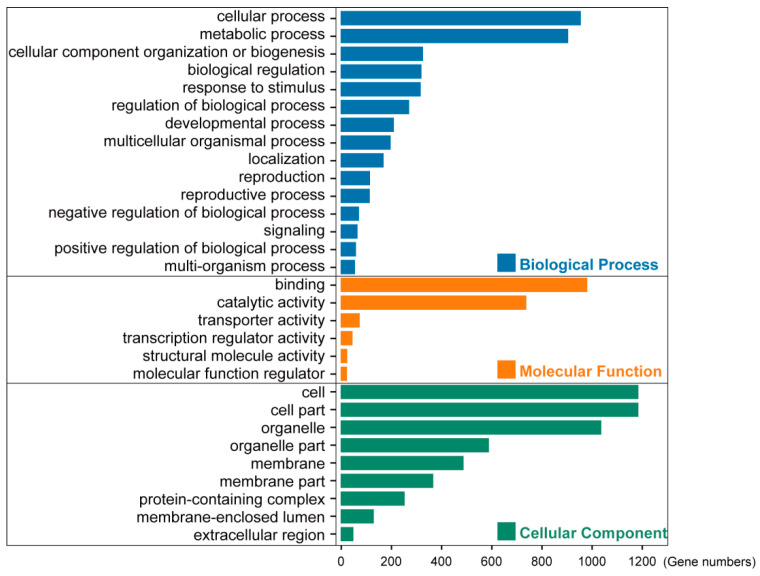
GO enrichment analysis 1499 genes in model plants *Arabidopsis thaliana* belonging to the 1785 OGs. The X-axis shows gene numbers, and the Y-axis lists the plant GO slim terms categories. Blue, orange, and green bars represent biological processes, molecular functions, and cellular components, respectively.

We used the dynamic GO enrichment analysis tool in OmicShare Gidio Bioinformatics cloud platform for GO enrichment analyses (https://www.omicshare.com/ (accessed on 28 April 2022)) (Figure 2). Ensemble_104 or 51 of Mode biology *Arabidopsis thaliana* (TAIR10) was selected as the background file.

### 2.4. Phylogenetic Analyses

**Figure 3:**
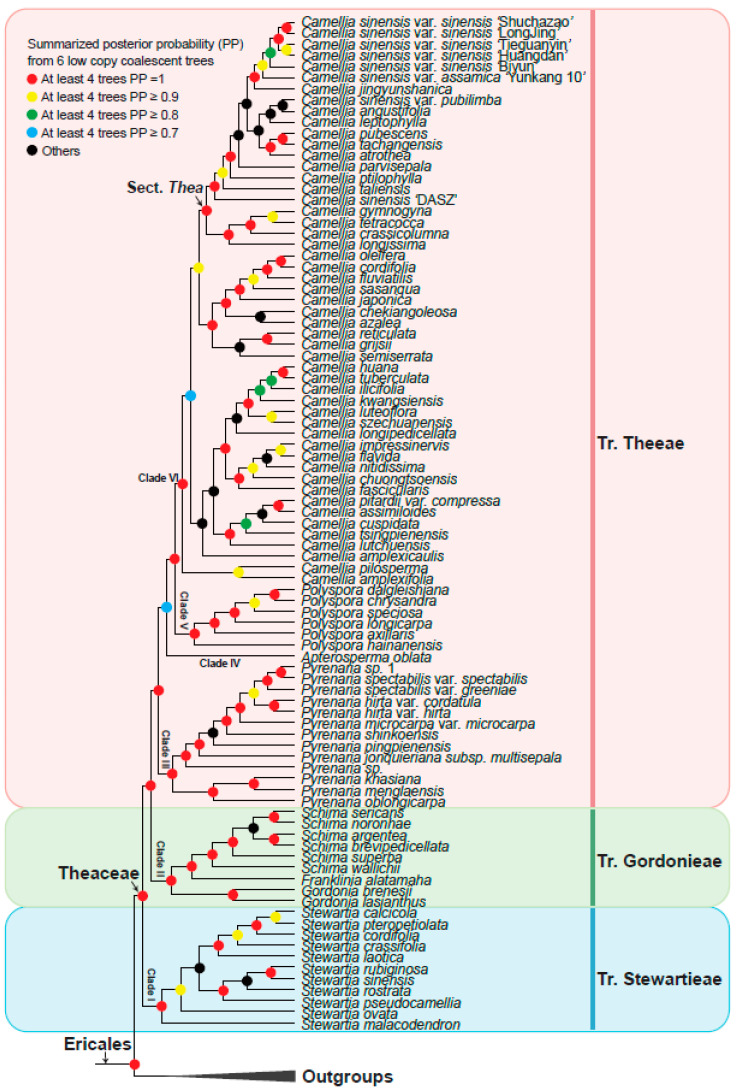
Summarized Theaceae phylogenetic relationships from six low-copy coalescent trees by ASTRAL. Colored labels at the nodes represent the summarized status of posterior probability (PP) in our six low copy coalescent trees: red circles for PP = 1 in at least four trees; yellow, green, and blue circles for ≥0.9 PP, ≥0.8 PP, and ≥0.7 PP in at least four trees, respectively; and black circles for other types. The pink, green, and blue vertical line on the right indicates Tr. Theeae, Tr. Gordonieae, and Tr. Stewartieae, respectively. The outgroups collapsed into a black triangle.

Phylogenetic trees were reconstructed based on low-copy orthologous genes. We obtained coalescent trees from six gene sets (gene sets 1–6) and summarized the topologies from the six coalescent trees to propose a final model tree (Figure 3). Amino acid sequences were aligned using MAFFT v7.487 [50] with the “–auto” parameter. Poorly aligned regions were further trimmed using trimAl v1.2 [51] software with the “-automated1” parameter. Multiple amino acid sequence alignments were converted to nucleotide alignments with PAL2NAL [52] software. Single-gene ML trees were reconstructed with IQ-TREE v2.1.4-beta [53] under the GTR+G model with 1000 bootstrap replicates. The coalescent analyses were implemented by Astral.5.7.8 [54]. We also concatenated the 253 OGs (sets 6) into a supermatrix and reconstructed the phylogenetic tree using IQ-TREE v2.1.4-beta [53] with 1000 bootstrap replicates under the GTR+G model.

In order to obtain a more accurate and high-supporting species relationship, multiple copies of orthologous genes were used for phylogenomic analyses. To reduce low-quality data, the assemblies with BUSCO completeness of less than 50% were excluded for further analyses. Finally, a total of 120 datasets proteins (including outgroups) were done “all against all” BLASTP by software DIAMOND [55]. The E value was 1 × 10^−5^ by default, and the other parameters were max-target-seqs 10,000 min-score 50, id 50, query-cover 75, subject-cover 75. Followed by clustering with the MCL algorithm (inflation index = 6.0) [56,57]. The OGs with at least five species and sequence lengths greater than or equal to 400 (set 7) were aligned using MAFFT v7.487 [50] with the ‘‘–auto’’parameter. Poorly aligned regions were further trimmed using trimAl v1.2 [51] software with the “-automated1” parameter. Next, additional filtering based on taxon coverage is more than 50%, alignment length is greater than 800 bp, yielded a gene set of 7429 OGs (set 8), and each OG was used to construct gene trees with the maximum likelihood method IQ-TREE v2.1.4-beta [53]. BS values were estimated from 1000 replicates using the GTR+G model. The software ASTRAL-Pro [58] was implemented to summarize these multi-copy gene family trees for a consensus species tree.

### 2.5. Divergence Time Estimation

Three fossils and two secondary calibration points were used to calibrate divergence time estimates. The assignments and ages of the fossils included crown group angiosperms 125~247.2 million years (Myr) [59], and stem group *Schima* 23.0~109 Myr with fruits and seeds fossil type. The earliest fossil tricoplate pollen (~125 Myr) associated with eudicots was assigned the minimal original age for the crown group eudicots [59,60]. Two secondary calibrations including stem group Theaceae 79.8~102.5 Myr and crown group Theaceae 39.6~74.7 Myr were adopted from a previous study [16]. The five fossil calibrations were implemented as the minimum constraint in our analyses. A Bayesian phylogenomic dating analysis of the 253 selected OGs genes with 422891 loci was performed in MCMCtree program from the PAML package [61]. The tree topology was confirmed to represent the inferences from our coalescence-based analysis of six low-copy nuclear genes from 128 samples, using the approximate likelihood calculation to determine branch lengths [62]. Molecular dating was conducted using an auto-correlated model of among-lineage rate variation, the GTR substitution model, and a uniform prior on the relative node times. Posterior distributions of node ages were estimated based on Markov chain Monte Carlo sampling, with samples drawn every 250 steps over 10 million steps, following a burn-in of 500,000 steps. We examined convergence by performing the analysis in duplicate, to ensure sufficient sampling. Date estimates were calibrated using fossil-based age constraints on five tree nodes.

### 2.6. Speciation Rate Calculation

In this study, BAMM (Bayesian Analysis of Macroevolutionary Mixtures) [63] was used to detect speciation rates across tea phylogeny. Priors were set according to values evaluated by the setBAMMpriors function in the R package of BAMMtools [63]. To eliminate the effect of non-randomness sampling in analyses, we specified a sampling probability file that provided the relevant sampling fractions of each terminal. We calculated the fractions scores by counting the number of species represented by each genus divided by the total number of that genus. The species richness data were obtained from Flora of China (http://www.efloras.org/ (accessed on 2 September 2021)) and a nomenclatural review of genera and tribes in Theaceae [64]. We sampled eight genera out of a total of nine genera of the tea family [64], including *Apterosperma*, *Camellia*, *Franklinia*, *Gordonia*, *Polyspora*, *Pyrenaria*, *Schima,* and *Stewartia* with species numbers 1, 280, 1, 40, 40, 26, 20, and 20, respectively. The MCMC simulation runs were performed for 50,000,000 generations under the speciation extinction model with sampling every 1000 generations. To be conservative, the first 20% of all samples were discarded as burn-in. The effective sample sizes were calculated to confirm the MCMCtree convergence. Results were then processed to detect the number and location of potential diversification-rate shifts and net speciation rate variation through time in BAMMtools [63].

## 3. Results

### 3.1. Identification of Low-Copy Orthologous Genes and Noise Filtrations

A total of 1785 low-copy orthologous nuclear genes were identified from 11 species using the OrthoFinder (v2.0.0) software (Figure 1A). Using the 1785 low-copy orthologous nuclear genes as seed genes, all species used in this study were searched using the HaMStR (v13.2.6) package [49]. For each seed in the same species, only one best-hit similar sequence was retained. Among the 128 publicly available datasets, an average of 82% of the seed genes can be found in each of the samples. Since the sequencing depth and integrity of de novo assembly in different transcriptomic datasets are different, in order to find more complete sequences in as many species as possible, we screened the coverage and average length of the 1785 genes when studying the phylogenetic relationship of Theaceae. Finally, a total of six gene sets of different sizes were obtained, and they were supposed to be effective in obtaining a stable and highly supported phylogenetic relationship of Theaceae (Figure 1B). In detail, a total of six low-copy nuclear gene sets (1785, 1419, 1154, 741, 283, and 253) were selected by the following steps: 1785 identified OGs were used as the largest gene set 1. For each gene in gene set 2 (1419 OGs), the gene can be found in at least 50% of the 128 samples, and the average length of the multiple sequence alignment after deleting sequences with the poorly aligned region is greater than 500 bp. For each gene in the gene set 3 (1154 OGs), the gene can be found in at least 70% of the 128 samples, and the average length of the multiple sequence alignment after deleting sequences with the poorly aligned region is greater than 700 bp. For each gene in the gene set 4 (741 OGs), the gene can be found in at least 80% of the 128 samples, and the average length of the matrix after deleting sequences with poor alignment quality is greater than 1000 bp. Both our studies and previous studies reported that Theaceae and its three tribes are monophyletic groups. The gene sets 5 (283 OGs) and 6 (253 OGs) were obtained from gene set 4 by keeping species in Theaceae and each of the three tribes Theeaes, Gordonieaes, and Stewartieaes as monophyletic groups, respectively.

### 3.2. Gene Ontology Analyses Suggested the Housekeeping Function for 1785 Low-Copy Nuclear Genes 

To obtain function annotation for these 1785 low-copy nuclear genes, we examined the gene ontology (GO) categories for those phylogenetic gene markers. The results showed the enrichment in biological process, molecular function, and cellular component (Figure 2). The top two groups with the largest number of genes were enriched in cellular process and metabolic process in biological process, binding and catalytic activity in molecular function, cell, and cell part in cellular components, respectively.

### 3.3. Theaceae Were Divided into Three Tribes: Theeae, Gordonieae, and Stewartieae

**Figure 4:**
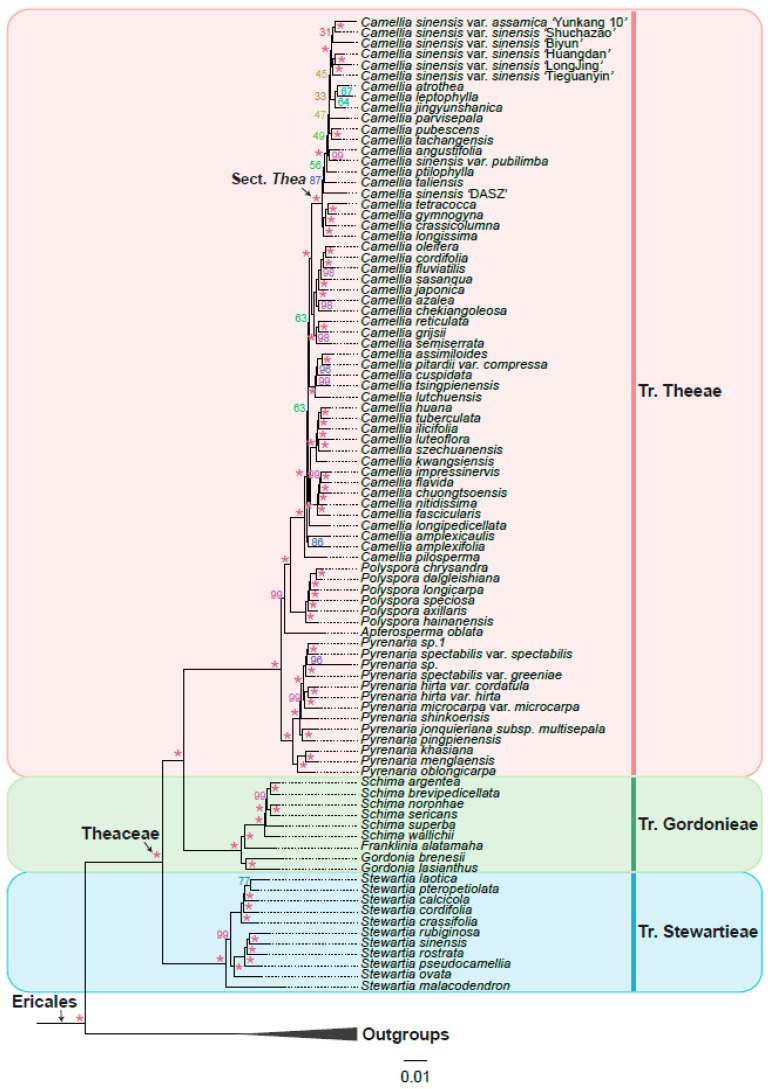
The maximum likelihood (ML) phylogenetic tree was inferred from a concatenated supermatrix of 253 low-copy nuclear genes. The ML tree was reconstructed using IQ-TREE with GTR+G model and 1000 bootstrap replicates. Asterisk indicates bootstrap support 100. The pink, green, and blue vertical line on the right indicate Tr. Theeae, Tr. Gordonieae, and Tr. Stewartieae, respectively. The outgroups collapsed into a black triangle.

**Figure 5:**
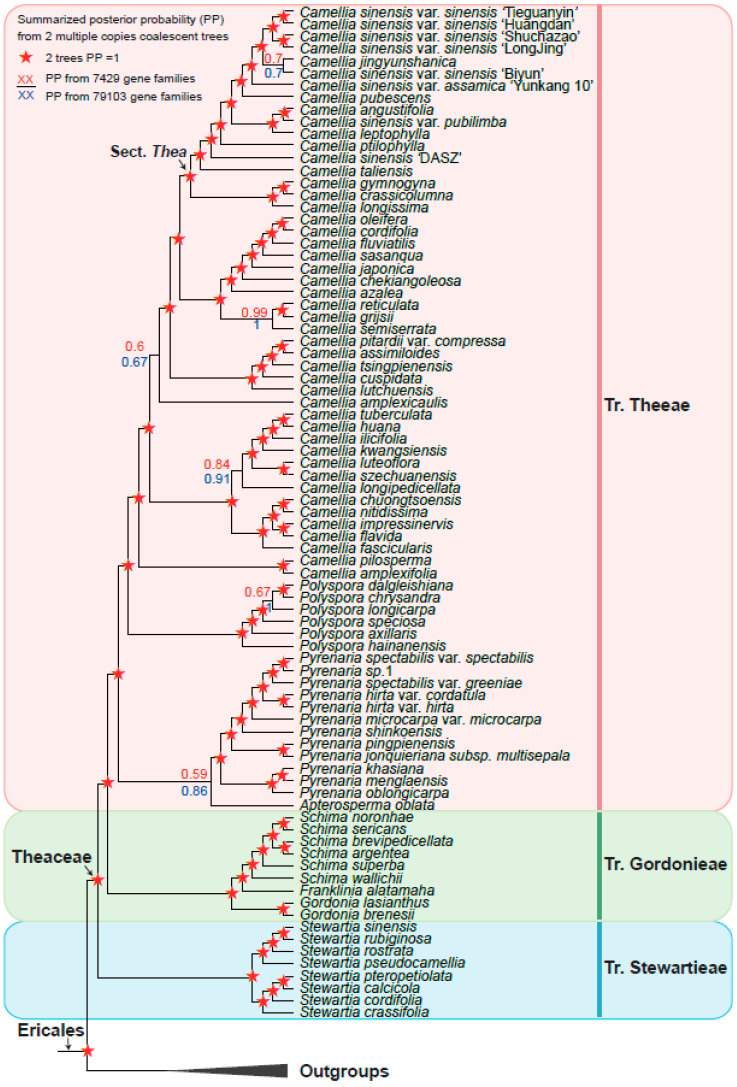
Summarized Theaceae phylogenetic relationships from two multi-copy coalescent trees by ASTRAL-Pro. Red pentagrams indicate maximal posterior probability (PP = 1) in two analyses. For nodes of other types, the node labels above (red) and below (blue) the branch are from 7429 and 79,103 gene families, respectively. The pink, green, and blue vertical line on the right indicate Tr. Theeae, Tr. Gordonieae, and Tr. Stewartieae, respectively. The outgroups collapsed into a black triangle.

Based on the six low-copy orthologous nuclear gene sets, we reconstructed a Theaceae phylogenetic tree on the 128 samples using the coalescent method (Figure 3). The six trees finally obtained high support and mostly consistent species relationships, with nearly 90% of the nodes having a posterior probability greater than or equal to 0.7 in at least four trees. Different gene sets showed that the Theaceae family was monophyletic, and the monophyly of the three tribes within the Theaceae family is well supported (six gene sets PP = 1). Our phylogenomic results strongly supported three monophyletic tribes belonging to six clades (Clades I–VI) in Theaceae (Figure 3). Our results consistently supported Stewartieae and Gordonieae as successive sister groups of Theeae (all gene sets PP = 1). To verify the reliability of the relationship, the maximum likelihood (ML) phylogenetic tree was also reconstructed from a concatenated supermatrix of 253 low-copy nuclear genes (Figure 4). The topology of three tribes in the supermatrix tree was completely consistent with the phylogeny from the coalescent method (BS = 100). The multi-copy nuclear gene coalescent trees were inferred from two gene sets with 120 samples (Figure 5). The multi-copy nuclear gene coalescent trees further confirmed that Stewartieae is the most divergent tribe, followed by Gordonieae and Theeae (PP = 1).

### 3.4. A Robust Topology at Generic Level of Theaceae

In this study, 128 samples representing 8 out of 9 genera of Theaceae were collected. Both the coalescence-based and concatenation-based methods (Figure 3 and Figure 4) supported the monophyly of each genus and generally consistent relationship of 8 genera.

The tribe Stewartieae (Clade I) includes 11 species of *Stewartia*; among Gordonieae (Clade II), *Gordonia lasianthus* + *Gordonia brenesii* were the first divergent lineage; the *Gordonia*, *Franklinia,* and *Schima* are successive sister groups of the tribe Theeae; Theeae (Clade III–Clade VI) is a tribe with the most species richness and the most complicated internal relationships among the three tribes. In Theeae, *Pyrenaria* (Clade III) with 13 representative species differentiated firstly; *Apterosperma* with one representative species is the second divergent lineage; and *Polyspora* with six representative species is the sister group of *Camellia* (at least four trees PP = 1 in Figure 3). The genus of *Camellia* with 51 representative species, including the large-leaf tea tree (CSA, *Camellia sinensis* var. *assamica*) and the small-leaf tea tree (CSS, *Camellia sinensis* var. *sinensis*), is the youngest lineage of Theaceae. Notably, *Camellia sinensis* is a polyphyletic group with three separated lineages including *Camellia sinensis* ‘DASZ’, *Camellia sinensis* var. *pubilimba,* and the combination of *Camellia sinensis* var. *sinensis* and *Camellia sinensis* var. *assamica* (Figure 3). Our phylogenetic results suggested that the classification of *Camellia sinensis* needs further revision.

However, in the phylogenetic relationship of *Camellia* based on the multi-copy nuclear genes of 120 samples (Figure 5), *Apterosperma* and *Pyrenaria* are sister groups to each other, with low support (PP = 0.59), which contradicts the coalescent phylogeny inferred from low-copy nuclear genes. Since we removed eight species with low assembly quality from multi-copy coalescent analyses, the phylogenetic relationship with a higher support value inferred from low-copy nuclear genes is more reliable [19].

### 3.5. Phylogeny Comparison from Inference by Low-Copy with Multi-Copy Nuclear Genes

The summarized coalescent phylogeny from six low-copy gene trees (LCGT, Figure 3) was compared with the coalescent tree of 7429 OGs multi-copy gene trees (MCGT, Figure 5); we found that they almost had the same topology except for only a few nodes (Figure 5). The differentiation between wild ancient tea plant *Camellia sinensis* ‘DASZ’ and *Camellia taliensis* is unclear. The *Camellia sinensis* ‘DASZ’ differentiated earlier than *Camellia taliensis* in LCGT, whereas *Camellia taliensis* differentiated earlier than *Camellia sinensis* ‘DASZ’ in MCGT. *Camellia jingyunshanica* and the other six *Camellia sinensis* (*Camellia sinensis* var. *sinensis* + *Camellia sinensis* var. *assamica*) genome species were sister groups in LCGT, which differentiated earlier than *Camellia sinensis* var. *assamica* ‘Yunkang 10′. In MCGT, *Camellia jingyunshanica* and *Camellia sinensis* var. *sinensis* ‘Biyun’ were sister groups and differentiated later than *Camellia sinensis* var. *assamica* ‘Yunkang 10′. In LCGT, *Camellia tsingpienensis* differentiated earlier than *Camellia cuspidata*, which is the opposite scenario in MCGT. *Pyrenaria jonquieriana* subsp. *multisepala* and *Pyrenaria pingpienensis* differentiated successively in LCGT. However, they were sister groups of each other in MCGT, which is consistent with a previous study in 2021 [19].

### 3.6. Molecular Dating Suggested Theaceae Originated Early Than the K-Pg Boundary

**Figure 6:**
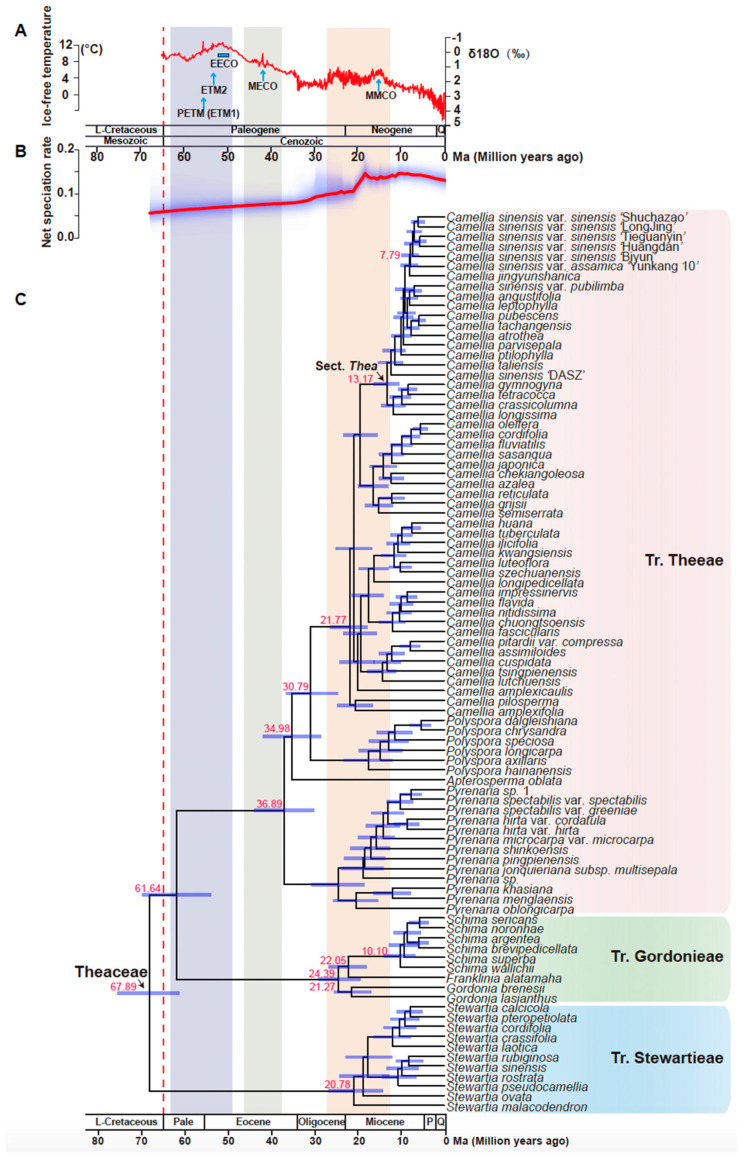
Chronogram of Theaceae estimated using a Bayesian relaxed molecular clock: (**A**) The global climate curve over the last 65 million years (modified from Zachos et al., 2008 [65] and Guo et al., 2020 [66]). Time periods of major climatic events are highlighted. EECO, Early Eocene Climatic Optimum; MECO, Mid-Eocene Climatic Optimum; MMCO, Mid-Miocene Climatic Optimum; PETM (ETM1), Paleocene Eocene Thermal Maximum 1; ETM2, Eocene Thermal Maximum 2. (**B**) Rate-through-time plot of Theaceae. The red line is the median diversification rate (species/million years) and the blue shadow indicates the 95% credibility interval. (**C**) A chronogram (timed phylogeny) of Theaceae. This time tree results from MCMCTree analysis using the model tree from six low copy coalescent trees. Red numbers represented node age. Tribes are listed on the right of the species’ names. The dashed red line indicates the Cretaceous-Paleogene (K-Pg) boundary. Light-blue bars indicate 95% credibility intervals of the divergence times. The geological timescale at the bottom is in million years. L-Cretaceous, Late Cretaceous; Pale, Paleocene; P, Pliocene; Q, Quaternary.

In our study, Bayes-based mole clock dating software MCMCtree [61] was used for divergence time estimation. The smallest gene set six of 253 selected OGs with 422891 loci was used to estimate branch length using a Bayesian method to obtain the evolutionary timescale of Theaceae lineages. A total of three fossils and two secondary calibration points were used for our dating analyses. As illustrated in the chronogram in Figure 6, the age of the stem and crown group of Theaceae were estimated at 82.4 and 67.9 million years ago (Mya), respectively (Figure 6). The three tribes diverged after a short period of ~6 million years from the most recent common ancestor (MRCA) of Theaceae. The divergence between tribe Gordonieae and Theeae was estimated at ~61.6 Mya. The crown group ages of Stewartieae, Gordonieae, and Theeae were estimated as 20.8, 24.4, and 36.9 Mya, respectively. The ages of the MRCA of the genus *Camellia* and section Thea were estimated at 21.8 and 13.2 Mya, respectively. The age estimation of Theaceae in our study is the general consensus with previous studies by different inference methods [18,19].

### 3.7. Camellia Specific Fast Radiation Associated with Climate Optimum

**Figure 7:**
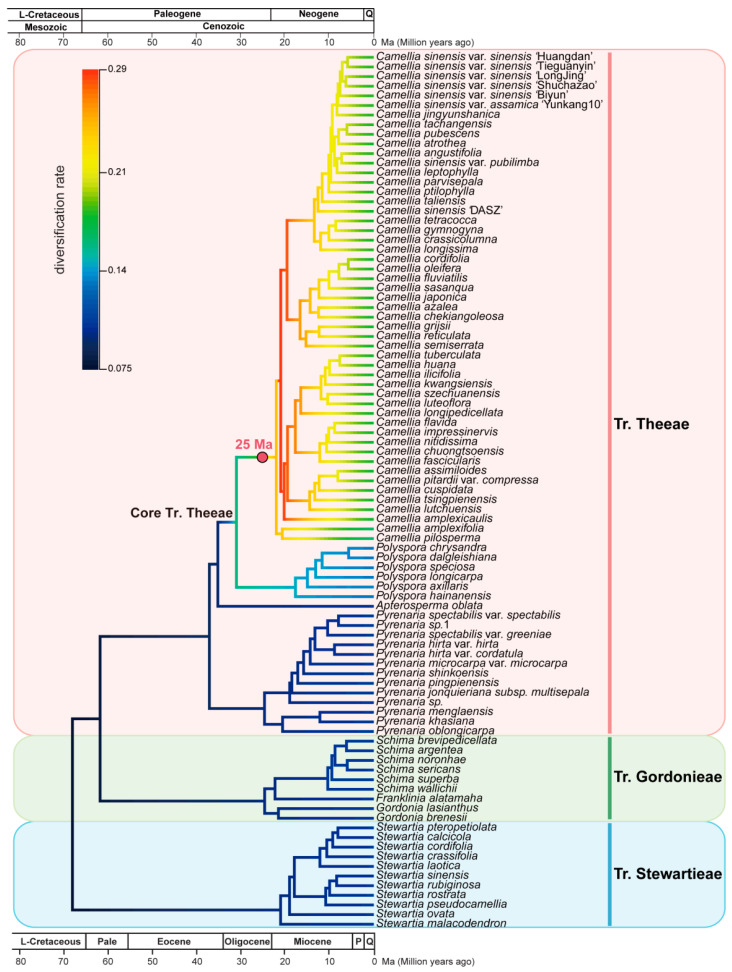
Diversification rate shifts in Theaceae. The red circle is species diversification rate shifts estimated by BAMM. The colors of branches show the mean diversification rate (species/million years) from BAMM. The geological timescale is illustrated at the top and bottom. The geological timescale at the bottom is in million years. L-Cretaceous, Late Cretaceous; Pale, Paleocene; P, Pliocene; Q, Quaternary. The pink, green and blue vertical line on the right indicates Tr. Theeae, Tr. Gordonieae and Tr. Stewartieae, respectively.

To investigate the species diversification dynamics in Theaceae, we used BAMM (Bayesian Analysis of Macro-evolutionary Mixtures) [63] to estimate diversification rates and the shifts in diversification rates using our time-calibrated phylogeny (Figure 6). We identified the speciation rate started acceleration from the Core Tr. Theeae in the late Oligocene at ~30.8 Mya (Figure 7). None of the diversification shift rates were detected in tribes Stewartieae and Gordonieae. Notably, only one significant diversification rate shift was detected in the MRCA of *Camellia*, a genus with the most species richness in Figure 7. Coincidently, this *Camellia*-specific diversification shift or a fast radiation event occurred associated with the Mid-Miocene Climatic Optimum (MMCO) according to the ancient ice-free temperature in Figure 6.

## 4. Discussion

Here, we used 1785 nuclear genes from 91 genomes and transcriptomes to reconstruct a robust Theaceae phylogeny, with a highly supported monophyly for three tribes and eight genera with two or more sampled taxa except for *Apterosperma oblata*. Furthermore, using the well-resolved phylogenetic relationships among Theaceae, we estimated their divergence times, investigated diversification dynamics, and analyzed the radiation of Theeae in adaptation to rigid environments.

### 4.1. Conserved Low-Copy Nuclear Genes with Main Housekeeping Functions Are Effective Markers for Theaceae Phylogeny

Low-copy nuclear genes have been widely used for phylogenetic analyses in different levels of taxons of plants, including main clades in Eudicot [25,67], family [24,36,38,66,68], and sub-families [66]. Although low-copy nuclear genes have been verified as gene markers for evolutionary analysis, their identification, representativeness, and qualities remain limited in Theaceae. Widespread whole gene duplication events hinder us from selecting effective true orthologous nuclear genes as phylogenetic markers [69]. Gene duplication makes it impedimental to distinguish orthologous genes from paralogs [70]. In some situations, single-copy paralogs resulting from gene duplication and subsequent lineage-specific losses could be mistaken as orthologs, leading to the incorrect inference of organismal relationships [19]. We herein integrated 91 genomes and transcriptome datasets representing species from nearly all groups of Theaceae. In order to identify low-copy nuclear genes, orthogroups containing one/two gene sequences from 11 angiosperms (at least one gene from each species) were obtained in our study. We identified 1785 OGs and GO analyses showed that most OGs were functionally conserved genes. Overall, identifying OGs in Theaceae provides significantly fundamental data to elucidate the phylogeny.

### 4.2. A Robust Theaceae Phylogeny Supported by Extensive Low-Copy and Multi-Copy Nuclear Gene Markers

Theaceae includes many significant woody species including tea plant, tea-oil tree, and *C.japonica*, which are used in the production of tea, edible oil, as well as ornamental flowers [2,5,71]. Previous studies on the phylogeny of Theaceae have mainly used chloroplast genes *rbcL* and *matK*, the mitochondrial gene *matR,* and the entire plastid genome sequences. Since the Theaceae has undergone at least two whole-genome duplication events and contains a large number of repetitive sequences [72], the chloroplast genes are rarely mutated compared with nuclear genes. Using many plastid genes still fails to obtain a resolved Theaceae phylogenetic tree. With the rapid development of high-throughput sequencing technology, more and more genomes and transcriptome data in Theaceae have been released, making it possible to resolve phylogenetic relationships using nuclear genes. In a recent study, the phylogenetic relationship of Theaceae has been reconstructed based on 610 low-copy nuclear genes by using the coalescent and supermatrix ML methods, respectively [19]. For the two different methods, Stewartieae and Gordonieae are successive sister groups of Theeae. However, the relationships within the genera are still not fully resolved and the location of *Apterosperma* is uncertain [19]. In our study, an evolutionary tree with the largest number of samples and nuclear genes was reconstructed to solve the complex phylogenetic relationship of Theaceae. A total of 91 Theaceae genome and transcriptome data were collected from the public, 1785 low-copy nuclear orthologous genes were identified, and the complex phylogenetic relationship of Theaceae was reconstructed based on the coalescent and supermatrix ML methods. Based on its morphological characters and phylogeny in our study, we speculated that the ancestor of Theaceae may be deciduous evergreen shrubs or small trees. Furthermore, 7429 multi-copies of homologous gene clusters from 120 samples after removing 8 samples with low genome completeness were used to reconstruct the complex phylogenetic relationship of Theaceae based on the coalescent method. A total of eight species of trees with generally the same relationship were obtained (Figure 3, Figure 4 and Figure 5), and consistently supported the Theaceae as monophyletic, while the three tribes were also monophyletic. We successfully resolved the phylogenetic relationship at the Theaceae family level. This study showed that Stewartieae and Gordonieae are successive sister groups of Theeae. It is clear that the earliest differentiation tribe is Stewartieae, followed by Gordonieae and Theeae. This is an evolutionary tree with the largest number of representatives and extensive nuclear genes, which was reconstructed to solve the complex phylogenetic relationship of Theaceae. 

In our study, the position of *Apterosperma* in the coalescent tree and supermatrix ML tree was consistent, and at least four in six coalescent trees have a support value (PP) greater than 0.7, and the support value (PP) of the supermatrix ML tree is 0.99. Based on our phylogenetic analyses from multi-copy nuclear genes, we believe that (([*Camellia*, *Polyspora*], *Apterosperma*), *Pyrenaria*) represents the possible relationship in tribe Theeae. However, the support for the recent common ancestor of the *Apterosperma* and *Camellia* is not very high. It is necessary to collect more species representatives of *Apterosperma* for further investigation. The relationships within the *Camellia*, the largest genus of the Theaceae family, have not yet completely been resolved. They are expected to be resolved with the completion of genome sequencing of more species and the development of sequencing technologies and computational methods. The genomes sequenced so far are mainly the eight species in *Camellia*, which were the most derived lineage compared with the entire Theaceae family. The released published genomes are relatively rare and are not efficient in providing enough data for phylogenetic analyses. None of the genome sequencings have been available for the species in the Gordonieae and the basal-most tribe Stewartieae. The transcriptome data provide limited gene content because their material is collected from specific tissues or cells in a particular developmental stage or stress state. It is hoped that more comprehensive phylogenetic relationships of the Theaceae can be obtained in the future by integrating more genome data representatives. Furthermore, the phylogenetic research of the Theaceae family is still full of challenges since the genome sequence of the basal group of the Stewartia has not yet been completed. Thus, comparative genomics research cannot yet be carried out, which limits the further study of this family, such as comparative and functional genomics. 

### 4.3. Genus Camellia Has a Significant Diversification Shift Rate Related to the Species Radiation with the Mid-Miocene Climatic Optimum

Paleoclimate profoundly influences the origin, extinction, biodiversity of species, and even ecosystem patterns [73]. The climate of the earth has undergone dramatic and complex changes ranging since the Cretaceous, including the hot periods of the Paleocene-Eocene Thermal Maximum (PETM), the Early Eocene Climatic Optimal (EECO), and the Middle Eocene Climatic Optimal (MECO), as well as the abrupt cooling during the E-O transition [65]. The genus *Camellia* has a significant diversification shift rate related to its species radiation with the Mid-Miocene Climatic Optimum (MMCO), which was first detected in this study. In the late Oligocene to early Miocene, the plate moved continentals frequently, which led to aridity and seasonal climates [74]. During this period, many new habitats were created, laying a foundation for the radiation of the genus *Camellia*. In the MMCO, the rapid differentiation of new species in the genus *Camellia* emerged. After the MMCO, there was another rapid cooling period to a low temperature. Key morphological innovations, such as the hardness and toughness of fruit skin, made them pull through these harsh environments. 

On the other hand, whole-genome duplication (WGD) is considered an important factor in developing stress resistance in plants [58,75]. The Theaceae has undergone the ancient WGD (Ad-γ), shared with other plants in core Eudicots [72,76]. The recent WGD event (referenced as Ad-β) that occurred in Theaceae was observed in the genome of *C. sinensis* var. *sinensis*, *C. taliensis*, *C. reticulata*, and *C. impressinervis* [26]. Genetic contribution of paleopolyploidy events of tea family possibly contributes to molecular innovation of *Camellia* for their adaptation to the extreme environment in the ancient difficult time. A WGD event together with massive segmental duplication events has potentially facilitated the expansion of gene families relevant to the abiotic stress tolerance [26,77].

The fruit consists of the skin and the seed. It is an organ of angiosperms that develops from the ovary or other parts of the flower (such as the receptacle, sepals, etc.) after the pistil is fertilized by pollination. The pericarp can be divided into exocarp, mesocarp, and endocarp, and is an important part of the sexual reproduction of plants. The structure of the pericarp not only affects the development of plant seeds [78], but also affects seed dispersal [79,80]. The sign of fruit ripening is pericarp and seed coat hardening. The various types of hardened pericarp structures provide protective barriers for seeds or fruits while allowing seeds to have a variety of propagation strategies. Pericarp hardening results from secondary wall cell deposition and lignification [81,82]. The shell of Theaceae, especially the genus *Camellia*, has a certain hardness and toughness, which is a biological barrier against adverse environmental conditions, pathogenic microorganisms, and herbivores [83].

### 4.4. A Fast Radiation Started from the Most Common Ancestors of Genus Camellia

The success of plant radiation depends on their adaptation to changing environments in different ways, including the fundamental changes in the forms of both gametophyte and sporophyte. The origin of the regulatory system could generate different structures for plants to protect themselves from the outside world. All these evolutionary dynamics depend on the variation in genome or gene sequences, which are also the genetic foundations of new species formation. The *Camellia* has many economic species, including tea plant (*Camellia sinensis*), oil-tea plant (*Camellia olifera*), and *Camellia japonica*. Besides its important economic value, the species in *Camellia* are the dominant elements of the subtropical evergreen broadleaved forests (EBLFs) in East Asia [21]. In our study, within Theeae, *Camellia* forms a 100% supported monophyletic group, and *Pyrenaria* and *Polyspora* are successive sister groups to *Camellia*. The branch length representing the nucleotide substitution rate within *Camellia* is much shorter than other lineages, implying that the most evolved genus is more accommodating to the environment (Figure 4). A recent study has also suggested that the East Asian Summer Monsoon (EASM) intensification in the late Miocene further triggered the species or population level diversification of specific lineages inhabiting the subtropical EBLFs in East Asia [16]. Compared with the number of Theaceae species in America (ca. 26) and tropical (ca. 87), the majority of the species diversity within the family occurs in subtropical Asia (ca. 148). Especially, the genus *Camellia* has a significant fast radiation event in subtropical Asia, with 98 of ca. 120 species.

## 5. Conclusions

We have reconstructed a robust phylogeny with three tribes and eight genera in Theaceae, and used hundreds of carefully selected low-copy nuclear genes and nearly ten thousand multi-copy nuclear genes to date. With a more representative sampling of Theaceae, we resolved the backbone relationship consistently across different phylogenetic methods. Our results generally agree with several earlier studies using plastomes, as well as nuclear genes, and provide strong support for the resolution of previously uncertain relationships, including that Stewartieae and Gordonieae are successive sister groups to the Theeae. Moreover, in the coalescent tree and the supermatrix ML tree, the position of *Apterosperma* is the same, which is differentiated after the *Pyrenaria*. (([*Camellia*, *Polyspora*], *Apterosperma*), *Pyrenaria*) represents the most likely genus relationship. We also present a well-resolved relationship of *Camellia*, which contains the vast majority of extant species richness of Theaceae. Here, we have shown that phylogenetically informative orthologous low-copy nuclear genes can be identified from large datasets and used to resolve the deep relationships of Theaceae. The well-supported topology in Theaceae provides a solid foundation for supporting further research, such as whole-genome duplication, functional evolution of development and physiology, biogeography, and species radiation. A well-supported tea plant phylogenetic tree can be used for mining wild germplasm resources, understanding the origin of economic species including tea trees and tea-oil trees with their wild relatives, and providing a phylogenetic reference map for the conservation of ecological diversity, ancient and endangered species, and the planning of nature reserves.

## Data Availability

None applicable.

## Supplementary Materials

The following supporting information can be downloaded at: https://www.mdpi.com/article/10.3390/biology11071007/s1, Table S1: Information of taxa included in this study and BUSCO assessment of assembly completeness.
